# Dietary seaweed extract mitigates oxidative stress in Nile tilapia by modulating inflammatory response and gut microbiota

**DOI:** 10.3389/fimmu.2024.1471261

**Published:** 2024-11-21

**Authors:** Muhammad A. B. Siddik, Prue Francis, Md Javed Foysal, David S. Francis

**Affiliations:** ^1^ Nutrition and Seafood Laboratory (NuSea.Lab), School of Life and Environmental Sciences, Deakin University, Queenscliff, VIC, Australia; ^2^ Department of Fisheries Biology and Genetics, Patuakhali Science and Technology University, Patuakhali, Bangladesh; ^3^ School of Environmental and Life Sciences, The University of Newcastle, Callaghan, NSW, Australia

**Keywords:** *Gracilaria tenuistipitata*, phenolic and flavonoid compounds, adipocyte tissue, temperature stress, RBC abnormality, tight junction protein, *Oreochromis niloticus*

## Abstract

**Introduction:**

Extreme water temperature affects the well-being of all aquatic animals, including fish. Higher temperatures can lead to the generation of reactive oxygen species (ROS), which can induce oxidative stress and negatively impact fish health and well-being. This study investigated the protective effects of seaweed extract on growth, antioxidant status, inflammatory responses, and gut microbiota to gain a better understanding of the acclimatization ability of Nile tilapia, *Oreochromis niloticus* in response to oxidative stress caused by high water temperatures.

**Methods:**

Red-seaweed, *Gracilaria tenuistipitata* rich in polyphenols (i.e., total phenolics and flavonoids content) was considered for the preparation of the *Gracilaria* extract (GE) for the study. Nile tilapia were fed the GE supplemented diet along with a control diet for 42 days, followed by 14 days of temperature ramping at a rate of 1°C every two days to the desired target (35°C) and 14 days of holding at 32°C for acclimatation.

**Results:**

Nile tilapia fed the GE had a significantly higher growth performance attributed to increased muscle fiber size compared to control (*p* < 0.05) after the 70 days of feeding trial. Fish fed the GE diet also showed a significantly lower lipid peroxidation by decreased malondialdehyde level when compared to control (*p* < 0.05). Furthermore, GE diet exhibited increased red blood cell counts with the decreased number of cellular and nuclear abnormalities. The gene expression of tight junction (i.e., *occludin, claudin1, ZO-1*) and *nrf2* (antioxidant biomarker) were upregulated, while *hsp70* (related to stress response) was downregulated in fish fed the GE diet. Additionally, GE supplementation led to an increase in bacterial diversity and the abundance of phylum Firmicutes, order *Lactobacillales*, and genera *Sphingobacterium* and *Prevotella* in the distal gut of Nile tilapia, which are mostly considered as beneficial for fish.

**Conclusion:**

The findings suggest that GE has the potential to be used as a dietary supplement to improve health, particularly as a stress-resistant supplement in the diet for Nile tilapia. This study may help make more informed decisions for tailoring the nutrient requirements of fish in the face of climate warming.

## 1 Introduction

Climate change has emerged as a serious threat in global aquaculture production, particularly due to changes in temperature patterns around the world (1). Increased water temperatures can result in the generation of reactive oxygen species (ROS), which could increase oxidative stress and negatively impact fish by disrupting their growth, reproduction, and overall wellbeing (2). Elevated water temperature is also known to result in a reduction of dissolved oxygen leading to hypoxia, causing further negative effects in the farmed fish (3). According to Huang et al. (1), every fish species has specific temperature ranges in which they thrive, where slight deviations from these ranges can negatively impact feed intake, growth, reproduction, metabolic activity, energy requirement, and utilization, as well as the overall fitness of ectothermic fish. Furthermore, acute and chronic stress resulting from temperature alterations have been shown to manifest in several metabolic (4), immunological (5), and neuroendocrinological (6) disturbances in fish. To uphold standard physiological performance, the inclusion of functional ingredients to aquafeeds is common place, with investigations into probiotics, prebiotics, functional amino acids, fatty acids, vitamins, and organic acids featured widely in studies evaluating the increase of temperature stress (7, 8). Moreover, the ability of seaweed-supplemented diets to strengthen the fish immune system and increase resilience to stress brought on by temperature has recently attracted the attention of the aquaculture industry.

Nutrient rich red-seaweeds such as *Gracilaria* sp. and *Asparagopsis* sp. have proven health benefits in fish (9, 10). Proteins, peptides, polysaccharides, and polyphenols are among the biologically active components in red-seaweeds, and their supplementation in aquafeeds have been reported to enhance growth, boost immune response, and increase disease resistance in fish (11). Notable polyphenols present in various red-seaweeds include phenolics, flavonoids, and carotenoids (12). These compounds are reported to have antioxidative, anti- inflammatory, and immunostimulatory effects when administered in animal nutrition (13, 14). Antioxidant properties in red-seaweeds help to neutralize harmful free radicals, reducing oxidative stress (15). Lowering oxidative stress may improve fish ability to cope with environmental stressors. For instance, Silva-Brito et al. (11) discovered that incorporating a 2.5% extract of *Gracilaria gracilis* in the diets of gilthead seabream, *Sparus aurata*, enhanced the immune response and reduced cortisol levels when they were subjected to the stress of overcrowding.

Red-seaweeds also contain specific carbohydrates (e.g., agar, agarose, and carrageenans) that can function as prebiotics in promoting host health (16). Prebiotics stimulate the growth of beneficial gut bacteria, which help maintain a healthy gut microbiome, promoting better health and improving disease resistance in fish. For example, Chen et al. (17) investigated the prebiotic effect of polysaccharides (e.g., total sugar, sulfate, and monosaccharides), extracted from red-seaweeds *Grateloupia filicina* and *Eucheuma spinosum*, which enhanced the growth of *Bifidobacterium*. Additionally, Ferreira et al. (18) reported that the inclusion of *G. gracilis* in the diet of European seabass, *Dicentrarchus labrax*, increased the abundance of *Sulfitobacter* and *Methylobacterium*. These bacteria are known to produce short- and medium-chain fatty acids that help reduce intestinal pH, playing a vital role in controlling the growth of pathogenic bacteria in fish (19, 20). In addition to red-seaweed, Zhang et al. (21) reported that dietary supplementation of green-seaweed, *Ulva pertusa*, in the diet of white-spotted rabbitfish, *Siganus canaliculatus*, led to an increase in the abundance of certain Firmicutes bacteria, notably *Ruminococcus*, *Clostridium*, and *Lachnospiraceae*. These bacteria play a key role in breaking down non-starch polysaccharides in the host gut, helping to maintain gut health, strengthen the intestinal barrier, and reduce the risk of intestinal inflammation, especially during times of stress.

The results mentioned above suggest that bioactive substances found in seaweeds have positive effects on immune function, especially in enhancing resilience against different bacterial diseases. Nevertheless, the impacts of seaweed-based diets to mitigate climate-induced stress such as temperature, have not been explored in aquaculture. Nile tilapia, *Oreochromis niloticus*, is one of the most commonly farmed fish across the world (22). Given the sensitivity of aquaculture to the effects of climate change, Nile tilapia could be assessed as a model species due to its higher thermal tolerance and wide distribution (23). A study conducted by Islam et al. (24) found that Nile tilapia had normal growth at 31°C but produce lower growth and physiological imbalance (i.e., erythrocytic cellular abnormalities and nuclear abnormalities) when cultured at high temperature (34°C). Therefore, the current study aimed to investigate the effects of dietary supplementation with red-seaweed (*Gracilaria* extract) on the potential mitigation of temperature-induced oxidative stress in Nile tilapia reared under high temperature. More precisely, it was examined how supplementing the feed of Nile tilapia with a *Gracilaria* extract containing bioactive compounds (i.e., total phenolic and flavonoid content) can regulate the immunological status and antioxidant response to cope at high temperature.

## 2 Materials and methods

### 2.1 Seaweed collection, processing, and experimental diet

**Figure 1:**
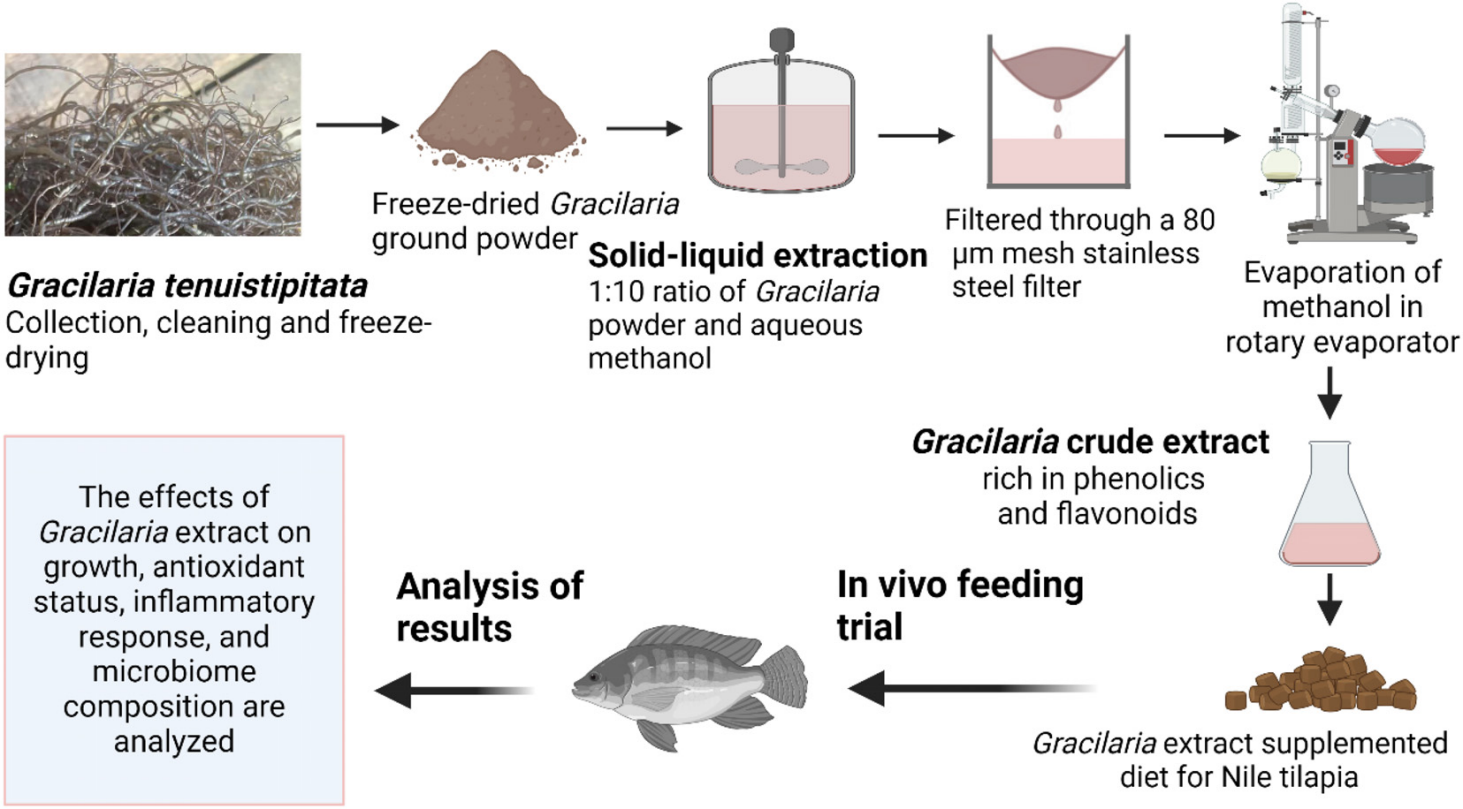
Production and utilization of *Gracilaria* extract from red-seaweed, *Gracilaria tenuistipitata*, for Nile tilapia.

*Gracilaria tenuistipitata* extract was prepared following the protocol described by Thépot et al. (25). The red-seaweed sample was collected in May 2022 from the Sonadia Sea Beach coast (21°29′N 91°54′E), Moheskhali, Bangladesh [water salinity ~32 ppt, temperature ~26°C] and thoroughly washed with tap water to remove unwanted contaminants (i.e., salt, sediments, sands, invertebrates, and epiphytes). The clean seaweed samples were then kept in a freeze drier at −80°C for 72 h. Once dried, the sample was grounded and sieved (200 µm mesh size) to produce a fine powder that was then vacuum-sealed and stored at −20°C for further processing. The *Gracilaria* extract (GE) was made with 70% methanol in a 1:10 (m:v) ratio under dark conditions about 12 h in each time. The crude extract was filtered (Whatman^®^ no. 2) and then evaporated slowly in a rotary evaporator (IKA^®^ RV3 Eco). Once the methanol had completely evaporated, the extract was then stored at −20°C for use. The various steps involved in making the GE are depicted in **Figure 1**. A representative amount of GE (triplicated) was dried using a freeze dryer at −80°C. The dried GE was then assessed for its nutrient composition (%) including protein (12.47 ± 1.57), lipid (1.35 ± 0.14), and ash (27.02 ± 1.50) based on a standard protocol (AOAC, 2023). The bioactive compounds, including total phenolic content (TPC) and total flavonoid content (TFC), were determined using the procedure described by Sobuj et al. (26). The results showed a TPC of 63.46 ± 1.77 mg GAE/g (gallic acid equivalents per gram) and a TFC of 29.71 ± 1.09 mg QE/g (quercetin equivalent per gram) using methanol as the solvent. The GE was added to the feed mixture along with all the other ingredients during the water addition step to make dough. Afterward, the dough was pelletized measuring 1.0–3.0 mm using a laboratory pelletizer. The pellets were dried at 50°C for 12 h and stored in airtight polythene bags in a refrigerator at 4°C until used. The feed formulation and chemical composition of the diet are presented in **Table 1**.

**Table 1:** Feed ingredients and proximate chemical composition of the basal Nile tilapia diet (% dry weight basis).

Feed ingredients	(%) composition
Fishmeal (anchovy) *	8.0
Soybean meal	32.0
Wheat flour	21.0
Wheat gluten	11.8
Rice bran	15.2
Vegetable oil	2.0
Fish oil	2.0
Starch	5.0
Lysine	0.5
NaCl	0.5
Mineral premix	1.0
Vitamin premix	1.0
Chemical composition (%) as fed basis	
Crude protein	33.16
Crude lipid	6.98
Crude fiber	6.22
Ash	7.75
Gross energy (kj g^−1^)	17.95

^*^Fishmeal: crude protein 65%, crude lipid 12%, moisture 7%, and ash 14%.

### 2.2 Fish and feeding trial

**Figure 2:**
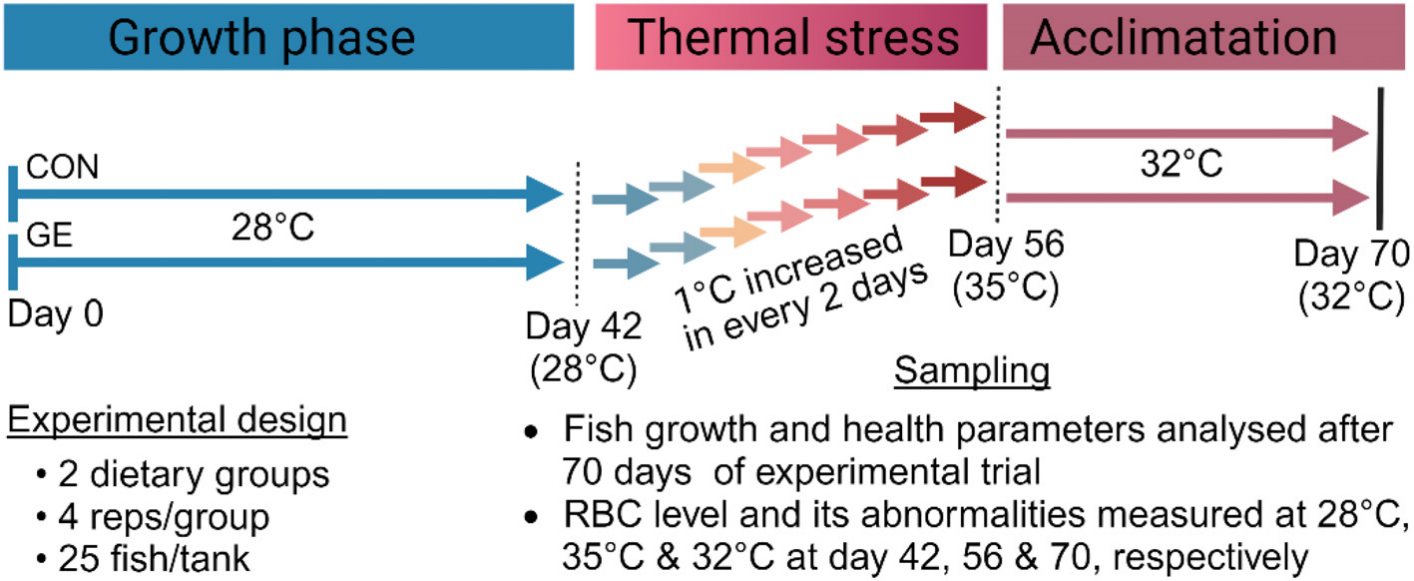
An overview of the experimental period chart.

Monosex Nile tilapia, *O. niloticus*, fry were purchased from a local fish hatchery (BRAC Fisheries, Khulna, Bangladesh). Nile tilapia fry were placed in eight experimental tanks, each with a 100-L water holding capacity, continuous aeration, and a water temperature of 28°C. They were then acclimatized in tanks for a period of 2 weeks where fish were fed the commercial tilapia diet (Quality Feeds Limited, Bangladesh) twice daily. After 2 weeks of acclimatization, fish were fasted for 24 h, then 200 similar-sized (1.61 ± 0.15 g) fry were randomly assigned into the same eight tanks divided into two dietary groups (four tanks per diet with 25 fish per tank). One quadruplicate group served as a control termed CON, while another group was fed the CON diet added with 1% *Gracilaria* extract (dry weight basis of feed) named GE. The concentration of GE (~1% of feed) was determined based on previous findings (27). The experiment duration was 10 weeks in which fish were kept under ambient temperature conditions (28°C) for the first 6 weeks. In the following 2 weeks, the water temperature was gradually increased by 1°C every 2 days from the initial temperature to 35°C to test the fish ability to withstand temperature changes. Subsequently, it was maintained at 32°C for 2 weeks to acclimatize the fish to a temperature 4°C higher than their original ambient temperature of 28°C. An overview of the experimental time chart is illustrated in **Figure 2**. Aerators were used in every tank to ensure enough oxygen (>5 mg/L). Fish were fed twice daily, at 08:00 and 16:00 h, until they appeared to be satisfied. The amount of feed delivered and the bulk weight of each replication tank were assessed every 2 weeks to monitor fish performance over time. To keep the water quality favorable for fish, regular siphoning was ensured to take out the unused feed and feces. Every morning, one-third of the tank water was replaced with temperature-controlled reservoir tank water to maintain the desired water temperature for fish.

### 2.3 Growth, survival, and feed utilization

Following the feeding trial, fish were counted and bulk weighed in each tank. Several performance parameters such as final body weight (FBW), specific growth rate (SGR), feed intake (FI), feed conversion ratio (FCR), hepatosomatic index (HSI), viscerasomatic index (VSI), and survival rate (SR) were calculated using the formula mentioned in an earlier study (28).

### 2.4 Histology of muscle, liver, and fat tissues

Eight randomly selected fish (two fish per each replicate) from each dietary group were sampled for liver and intraperitoneal fat histology. The collected samples were cleaned with normal saline solution to remove blood and other unwanted substances and were preserved immediately in 10% buffered formalin and stored at 4°C until further processing. The muscle and fat samples were preserved in 70% alcohol at 4°C until histological analysis. Assessment of lipid accumulation in the liver was performed using Oil Red O staining, whereas muscle and intraperitoneal fat samples were stained with hematoxylin and eosin (H&E). The histology was performed based on standard protocol. Briefly, fixed samples were dehydrated by passing through a graded series of alcohol. After dehydration, the samples were molted with wax and kept in cool storage. The samples were then sectioned (5 μm) using a microtome. The sectioned tissues were stained with H&E and observed under a light microscope (Olympus, Germany) at 400× magnification with photos captured with an onboard camera (BX40F4, Olympus, Tokyo, Japan) connected to the microscope. Muscle microstructure such as fiber diameter and density were assessed by nonparametric statistical procedures according to Rowlerson et al. (29). The adipocyte number and diameter were determined from 30 intact cells from each dietary group according to Chaklader et al. (30). All images were captured using a light digital microscope connected to a camera and ImageJ software (version: 1.53). The protocol used to calculate lipid droplet accumulation in the liver was described in **Supplementary Figure S1**).

### 2.5 Serum biochemical responses

At the end of the feeding trial, 4 fish from each replicate tank (16 fish/dietary group) were chosen at random, and blood samples were taken by puncturing the caudal vein and kept in non-heparinized tubes, left to settle for 6 h, and then centrifuged at 4°C for 10 min to extract serum that was kept at −20°C. The lysozyme activity of serum was analyzed following Siddik et al. (31). The levels of aspartate aminotransferase (AST), alanine transaminase (ALT), cholesterol, triglycerides, and glycogen were analyzed by an automated blood analyzer (SLIM; SEAC Inc., Florence, Italy) following Blanc et al. (32).

### 2.6 Antioxidant response

Fish that were being considered for blood collection were dissected to obtain liver tissue for measuring the antioxidant response. The activities of antioxidant enzymes including malondialdehyde (MDA), superoxide dismutase (SOD), and catalase (CAT) were assessed using commercial kits from ZellBio GmbH, Lonsee, Germany.

### 2.7 Real-time quantitative PCR

Total RNA from frozen hind gut samples of GE and control-fed fish was isolated using RNeasy Mini Plus Kit (Qiagen, Hilden, Germany) following the manufacturer’s instructions. The quality and quantity of the RNA were evaluated through agarose gel electrophoresis and spectrophotometry (NanoDrop^®^ ND-2000), respectively. cDNA synthesis was performed using a PrimeScript™ RT reagent kit (Takara, Japan). The real-time quantitative PCR (RT-qPCR) primers used in this study for Nile tilapia are listed in **Table 2**. The melting curve of the amplicon from the primers was obtained using uMELT software. Gene expression levels were measured by RT-qPCR with PowerUp™ SYBR Green Master Mix (Thermo Scientific, USA) on the 7500 Real-Time PCR System (Applied Biosystems, USA). The following conditions were used for real-time PCR: initial denaturation for 2 min at 95°C, followed by 40 cycles of amplification stating 30 s denaturation at 95°C, annealing for 1 min at 60°C, and extension at 72°C for 30 s. The melting stage in PCR began with the 95°C heating step for 15 s and cycled to 70°C cooling for 1 minute, with the continuous increase of 0.015°C per second. The analysis of RT-qPCR data for relative gene expression levels was normalized to the β-actin content in each sample and quantified using the 2^−ΔΔCt^ method as outlined by Livak and Schmittgen (33).

**Table 2:** The list of primer sequences utilized for RT-qPCR analysis.

Target genes	Primer sequences (5′ –3′) (F: Forward, R: Reverse)	Target size (bp)	Annealing temp. (°C)	Efficiency (%)	Reference
*il-1β*	F: GACAGCCAAAAGAGGAGC R: TCTCAGCGATGGGTGTAG	95	61	99.66	KF747686.1
*tnf-α*	F: CCAGAAGCACTAAAGGCGAAGA R: CCTTGGCTTTGCTGCTGATC	119	59	87.63	AY428948.1
*ZO-1*	F: CCGCAGATCAGTCCCTCTTC R: GTACGGAGTTAGCATCGCCA	134	67	88.45	XM_013270540
*occludin*	F: GGAGGAAAGCCGCAGTGTTCAG R: GTCGTAGGCATCGTCATTGTAGGA	109	62	89.45	XM_025899615.1
*claudin1*	F: GTCTGTTTCTGGGCGTGGTGTC R: ACTCCGACTGACTCCTCATCTTCC	135	60	88.61	XM_019367708.2
*nrf2*	F: CTGCCGTAAACGCAAGATGG R: ATCCGTTGACTGCTGAAGGG	40	62	92.38	XM_003447296.
*hsp70*	F: TGGAGTCCTACGCCTTCAACA R: CAGGTAGCACCAGTGGGCAT	85	59	94.17	FJ213839.1
*β-actin*	F: AGCAAGCAGGAGTACGATGAG R: TGTGTGGTGTGTGGTTGTTTTG	143	60	95.31	KJ126772.1

### 2.8 Blood RBC level and cellular and nuclear abnormalities

Blood red blood cell (RBC) level and its cellular and nuclear abnormalities in fish were measured in Nile tilapia at three different temperatures across the feeding trial in D42 (pre-temperature stress at 28°C), D56 (post-temperature stress at 35°C), and D70 (post-temperature adaptation at 32°C). Two fish from each replicate tank were selected for blood sampling and euthanized after recording their weights for biomass calculations. RBC counts were determined using a hemocytometer. The blood smear slides were prepared immediately after collecting the blood. The blood was spread along the edge of the slide and tilted at a 45° angle to create a thin, even smear. After air-drying for 5 min, the smear was fixed in methanol for approximately 2 min. Once dry and fixed, it was stained with a 5% Giemsa solution. The slides were then air-dried overnight and mounted with dibutylphthalate polystyrene xylene (DPX). The samples were examined under an optical microscope (G-206, Italy) using a 100× objective lens to assess RBC cellular and nuclear abnormalities. RBC cellular abnormalities were categorized as elongated (significantly longer than wider), fusion (joining of more than two cells to create a larger mass), and twin (two cells connected by their surfaces). RBC nuclear abnormalities consisted of micronucleus (circular chromatin bodies resembling the central nucleus), nuclear bridge (a strip of nuclear material connecting two nuclei within separate erythrocytes or within a single erythrocyte), and nuclear degeneration (nuclear condensation and extrusion leading to the creation of a pyrenocyte structure, which is subsequently engulfed and broken down by macrophages).

### 2.9 Amplicon sequencing

Eight fish from each dietary treatment were randomly selected to obtain hind gut samples, following appropriate biosafety measures. The gut samples, which included mucosa and digesta, were homogenized using a TissueLyser II (Qiagen, Hilden, Germany). Genomic DNA extraction was then carried out using the DNeasy PowerSoil Pro Kit (Qiagen, Hilden, Germany) following the instructions provided by the manufacturer. The DNA was quantified using a NanoDrop 2000c (Thermo Fisher Scientific, USA), and its quality was assessed by running it on a 1% agarose gel. To prepare the final master mix, 50 μL of Hot Start 2× Master mix (New England BioLabs Inc., USA), as per the manufacturer’s instructions, was combined with 2 μL of template DNA, 1 μL each of V3V4 primers (10 µM) with Illumina overhang adapter, and 21 μL of nuclease-free treated water. The mixture was then subjected to 35 cycles of amplification using a BioRad S1000 Thermal Cycler (BioRad Laboratories Inc., USA). Positive amplicons were purified using AMPure beads and indexed according to the Illumina 16S Sequencing Library Preparation protocol (Part # 15044223 Rev. B). Finally, the equimolar amplicons were pooled and sequenced on an Illumina MiSeq platform (Illumina Inc., San Diego, California, USA) using the paired-end, v3 kit with 600 cycles.

### 2.10 Processing of Illumina data

Paired-end amplicon sequence data (gz format) were imported in qiime2 (v2021.11) for further processing. The denoising of reads was performed using DADA2 followed by trimming of demultiplexed reads with parameters such as -p-trim-left-f l0; -p-trunc-len-f 260; -p-trim-left-r 10; -p-trunc-len-r 220. Chimeric sequences with >0.05% error rates were removed and non-chimeric reads (82%) indicating biological features were tabulated for feature frequency amplicon sequence variants (ASVs). The lowest non-zero frequency of 10 was set to filter the feature ASV table. Phylogenetic classification of ASVs into different taxa levels was performed using the qiime2 “classify consensus- blast” plug-in against SILVA 138 release (34). The feature table collapsed with taxonomy and subsequently removed mitochondrial and chloroplast sequences (<1% of reads). There were variations in *Clostridium* classification, and as a result, these variations were classified as “*Clostridium* sensu stricto 1-9” throughout the dataset. To make a homogeneous dataset for *Clostridium*, we renamed all “*Clostridium* sensu stricto 1-9” as “*Clostridium*”, and other non-classified reads into the “Unclassified” bacterial group. The final set of data was normalized (also called “rarefaction”) at an even depth of 15,236 for bacterial community analysis. Metagenome prediction of functional features from 16S rRNA data was performed with the PICRUSt2 pipeline (35).

### 2.11 Diversity and composition analysis

The ASV table, taxonomy, and metadata files were imported into R software (v4.22) for diversity and composition analysis (36). The number of shared and unique taxa were calculated using the MicEco package (37). Species richness (observed), Chao1, Shannon, and Simpson diversity were considered for alpha-diversity measurements. Beta-diversity was performed as a UniFrac distance metric (Unweighted and Weighted) and PERMANOVA was conducted for the visualization of feeding effects on beta-dispersion with 1,000 permutations using vegan and phyloseq (38) R packages. The distance between samples for a group was calculated as the “Bray–Curtis “ distance. We considered 1% taxa abundance per sample as a threshold for composition analysis at the phylum and genus level (39). Further analysis of differential abundance between groups was performed as Linear Discriminant Analysis (LDA) using the MicrobiomeMarker R package (40). An LDA cutoff value of 2.0 and a *p*-value of <0.05 were considered as statistically significant for compositional difference analysis.

### 2.12 Statistical analysis

Data were assessed for normality using the Shapiro–Wilk test and then analyzed with two-tailed Student’s *t*-tests and two-way analysis of variance (ANOVA). Two-tailed Student’s *t*-tests were applied to compare the effects of GE diet to control. The total RBC count, along with its cellular and nuclear abnormalities, was analyzed using two-way ANOVA. Additionally, alpha-diversity, beta-diversity, and compositional log-fold changes between the two dietary groups in microbiome composition were analyzed using two-tailed *t*-tests. Gut microbiome analysis was carried out using the R software. A *p*-value of <0.05, <0.01, and <0.001were considered statistically significant between the two dietary groups of GE and CON.

## 3 Results

### 3.1 Growth performances, feed utilization, and muscle health

**Figure 3:**
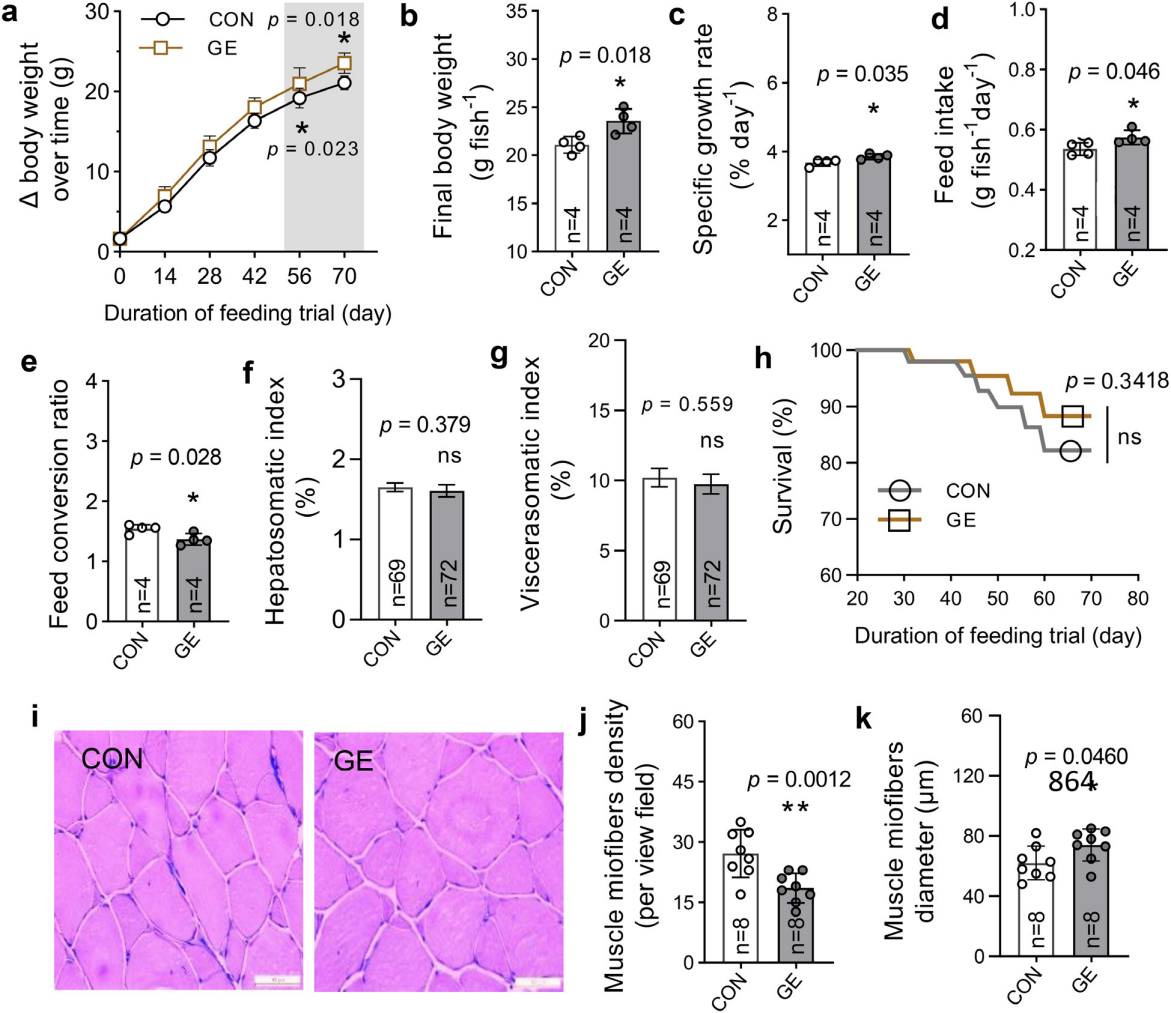
Growth and muscle health of Nile tilapia fed *Gracilaria tenuistipitata* extract for 70 days. **(A–H)** Growth performance, feed utilization, somatic indices, and survival of fish. **(I)** Transverse section of dorsal muscle microstructure (×200, H&E). **(J, K)** The myofiber development in fish in terms of fiber diameter and density. The data are presented as mean ± SD. Asterisks * and ** above the bars indicate significant differences between the two dietary groups of GE and CON, as determined by an unpaired *t*-test at *p* < 0 *<*.05 and *p* < 0.01, respectively. ns, non-significant; GE, *Gracilaria* extract; CON, control.

Nile tilapia fed the GE diet showed a significant improvement in growth performance and feed utilization compared to the CON (**Figures 3A–E**, *p* < 0.05). However, the somatic indices such as hepatosomatic index and viscerasomatic index were not influenced by the GE supplementation (**Figures 3F, G**, *p* > 0.05). Additionally, there was no significant difference in the survival rate between the two dietary groups (**Figure 3H**). The higher growth performance was supported by the enhanced dorsal muscle fiber diameter in fish fed the GE diet (**Figures 3I–K**).

### 3.2 Liver health and adipocyte distribution

**Figure 4:**
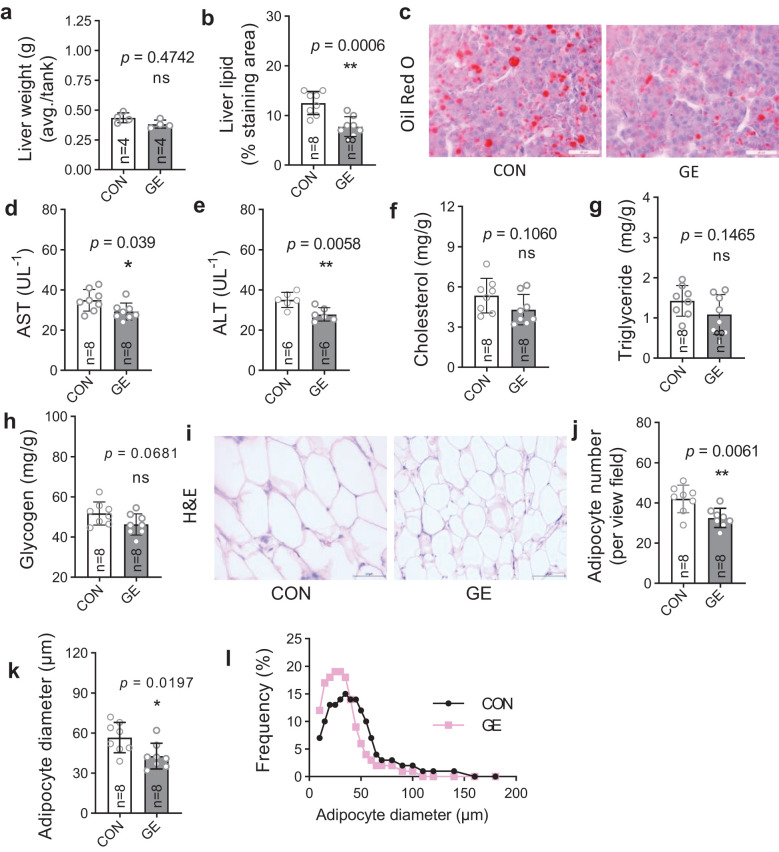
Liver health and intraperitoneal fat distribution in Nile tilapia fed *Gracilaria tenuistipitata* extract in varying water temperatures for 70 days. **(A, B)** Liver weight and quantification of lipid droplet area in liver of Nile tilapia. **(C)** Representative Oil Red O-stained liver histology showing lipid droplets (*n* = 8, magnification 50×). **(D-H)** Quantification of serum AST, ALT, cholesterol, triglyceride, and glycogen levels as liver health indicator. **(I)** Histomorphometry of intestinal adipocytes (*n* = 8, 10 fields per section, magnification 50×, H&E). **(J–L)** Quantitative image analysis for adipocyte distribution in terms of average adipocyte numbers, diameter (μm), and frequency (%). Data are the mean ± SD (standard deviation). An unpaired *t*-test is used to compare the results from GE-fed fish to control at *p < 0.05 and **p < 0.01. ns, non-significant; GE, *Gracilaria* extract; CON, control.

Hepatic lipid droplets significantly reduced in the GE-fed fish compared to the control, while liver weight exhibited no significant difference between the dietary groups (**Figures 4A–C**). Likewise, the hepatic enzymes AST and ALT were lower in GE-fed fish compared to control-fed fish, whereas cholesterol, triglyceride, and glycogen levels were not affected by seaweed supplementation (**Figures 4D–H**). Adipocyte numbers and fatty tissue diameter in fish fed the control diet were significantly decreased in comparison to fish fed the control diet (**Figures 4I–K**). The frequency of adipocyte diameter increased in GE-fed fish up to 50 μm, while adipocytes in fish fed the CON diet were greater in diameter (>50 to 200 μm) (**Figure 4L**).

### 3.3 Antioxidant status, immunity, and gene expression

**Figure 5:**
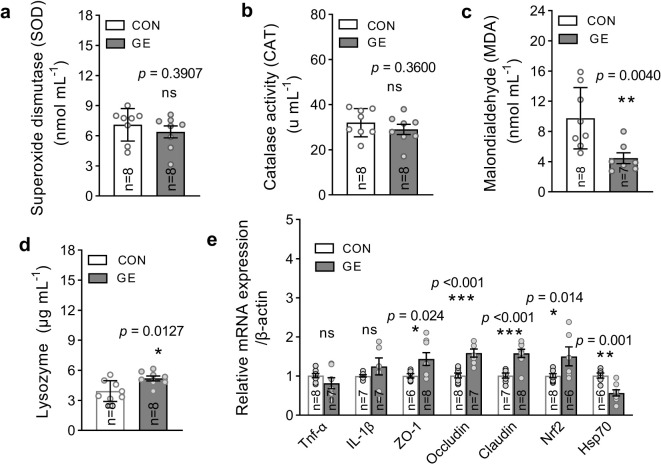
Antioxidant enzymes, lysozyme activity, and mRNA expression of Nile tilapia fed a diet supplemented with *Gracilaria tenuistipitata* extract in varying water temperatures for 70 days. **(A–C)** Quantification of oxidative stress through MDA, SOD, and CAT activities in fish liver after 10 weeks of feeding trial. **(D)** Immune response in terms of lysozyme activity in blood serum. **(E)** The relative quantification of mRNA expression of pro-inflammatory cytokines (*tnf-α* and *il-1β*), antioxidant gene (*nrf2*), tight junction genes (*ZO-1*, *occludin*, and *claudin1*), and heat shock protein 70 (*hsp70*) in the hind gut of fish. The data are presented as mean ± SD. Asterisks (*, **, and ***) indicate significant differences between the two dietary groups, control, and GE (*Gracilaria* extract), as determined by an unpaired *t*-test at *p* < 0 *<*.05, *p* < 0 *<*.01, and *p* < 0 *<*.001, respectively. ns, non-significant; GE, *Gracilaria* extract; CON, control.

The level of MDA significantly reduced in fish fed the GE-supplemented diet while CAT and SOD levels exhibited no difference between the two dietary groups (**Figures 5A–C**, *p* < 0.05). The lysozyme activity was enhanced in the GE group compared to the CON group (**Figure 5D**). Dietary GE supplementation significantly upregulated the expression of tight junction proteins (*occludin*, *claudin1*, and *ZO-1*) and the antioxidant gene, *nrf2*, while a downregulation of *hsp70* was observed in the GE group compared to the CON group. The pro-inflammatory cytokines *tnf-α* and *il-1β* were found unaffected by seaweed supplementation (**Figure 5E**, *p* > 0.05).

### 3.4 Blood RBC level and cellular and nuclear abnormalities in response to temperature stress

**Figure 6:**
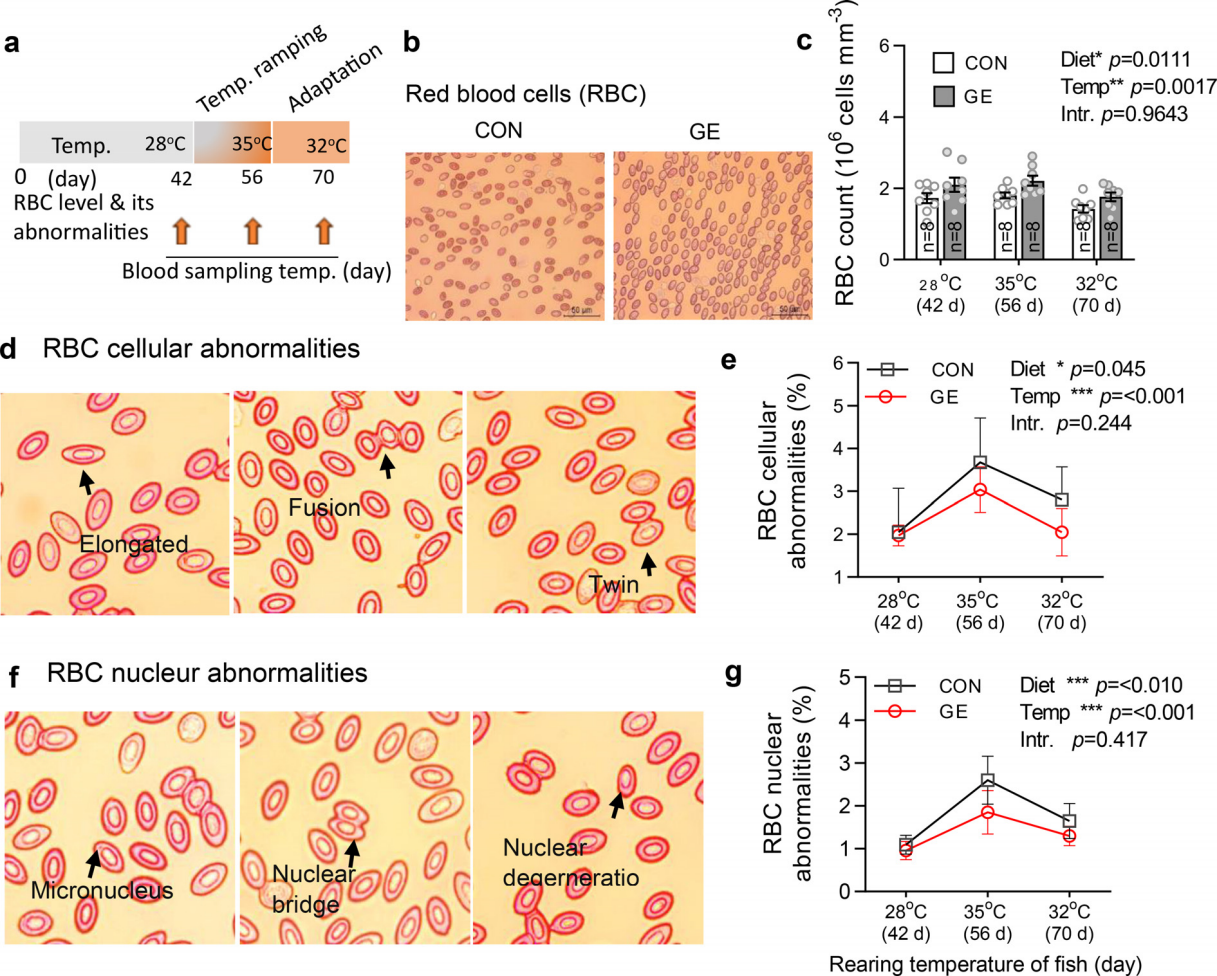
Effects of *Gracilaria* extract (GE)-supplemented diet on blood RBC level and its cellular and nuclear abnormalities in Nile tilapia in varying temperatures of 28°C, 35°C, and 32°C at days 42, 56, and 70, respectively. **(A)** Timeline of blood sampling at various temperatures during the feeding trial. **(B, C)** RBC level and its cellular and nuclear abnormalities in fish at 42 days (pre-temperature stress at 28°C), 56 days (post-temperature stress at 35°C), and 70 days (adaptation at 32°C). **(D, E)** representative H&E-stained blood histology showing RBC cellular abnormalities (elongated, fusion, and twin) and their quantification in GE and CON fed fish. **(F, G)** RBC nuclear abnormalities (micronucleus, nuclear bridge, and nuclear degeneration) and their quantification in fish fed GE and CON. Data are presented as mean ± SD. Asterisks (*, **, and ***) indicate significant differences between dietary groups and temperatures, determined by two-way ANOVA at *p* < 0.05, *p* < 0.01, and *p* < 0.001, respectively. GE, *Gracilaria* extract; CON, control; Temp, temperature; Intr, interaction.

RBC count and cellular and nuclear abnormalities were found significantly different by both the dietary groups and temperatures (**Figure 6**, *p* < 0.05). Fish fed the GE-supplemented diet significantly enhanced the RBC level when compared to the control. Likewise, the total frequencies of RBC cellular and nuclear abnormalities were found significantly lowered in the control group when compared to the control. However, fish at 35°C post-temperature stress produced more cellular and nuclear abnormalities compared to pre-temperature stress and post- temperature adaptation at 28°C and 32°C, respectively.

### 3.5 Sequence statistics and diversity of gut microbiota

**Figure 7:**
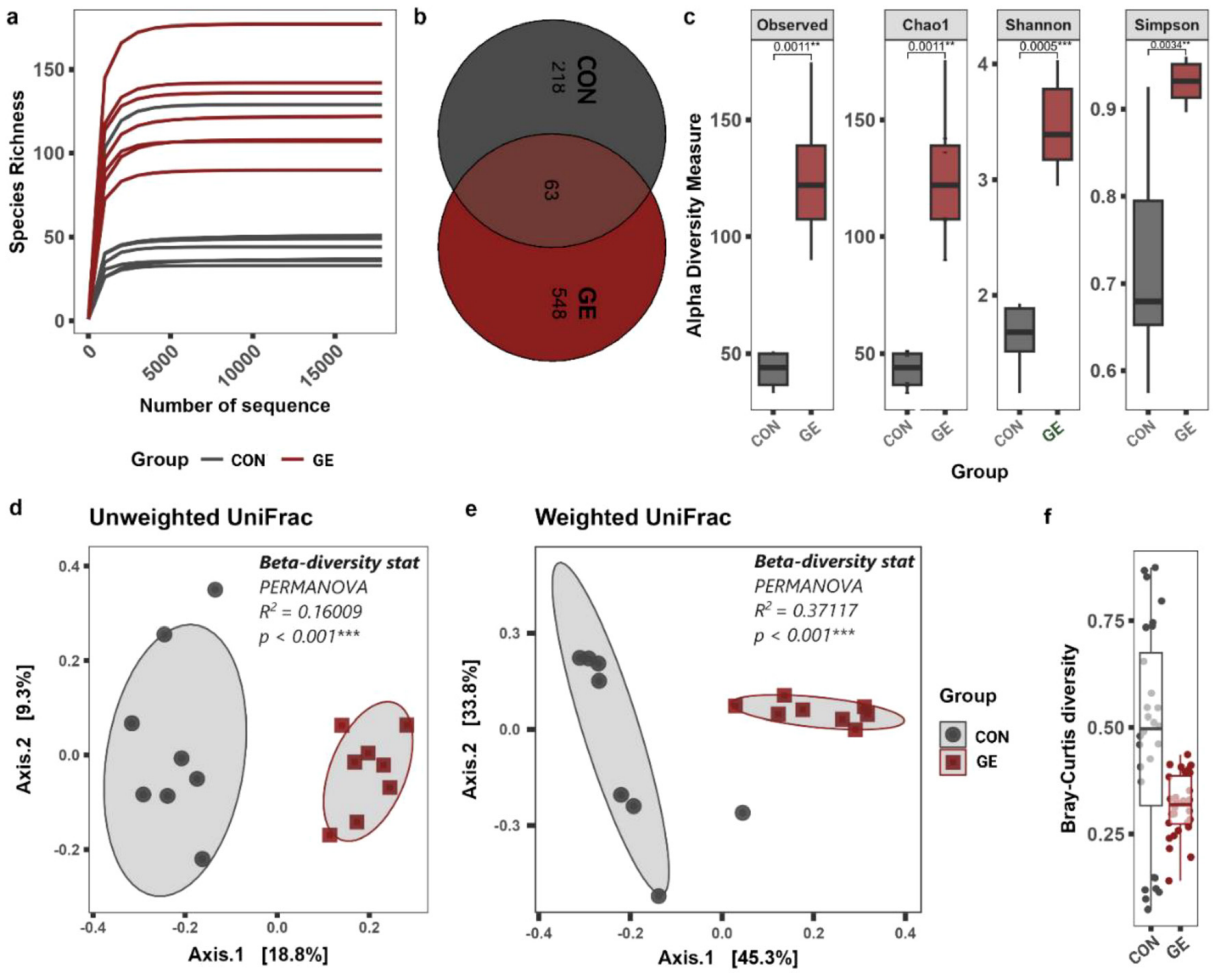
Effects of seaweed extract-supplemented diet on alpha-/beta-diversity of gut microbiota in Nile tilapia. **(A)** Rarefaction curve showing the depth and saturation level of study samples. **(B)** Number of shared and unique taxa. **(C)** Alpha-diversity measurements in terms of observed species, Chao1, Shannon, and Simpson diversity index. **(D, E)** Beta- diversity PCoA plot based on unweighted and weighted UniFrac distance matric. **(F)** The normalized pattern of Bray–Curtis distance represents the patterns of community distributions for CON and GE diets. Asterisks (** and ***) above the bars indicate significant differences between the two dietary groups of CON and GE determined by an unpaired *t*-test at *p* < 0 <.01, and *p* < 0 <.001, respectively. GE, *Gracilaria* extract; CON, control.

A total of 322, 761-bp quality reads were obtained after trimming, with an average of 20,172.6 ± 941.7 and ranging from 16,860 to 30,244 bp that were classified into 1,503 ASVs, six phyla, and 455 genera. The average good’s coverage index value of 0.998 and plateaued rarefaction curve were observed, indicating adequate depth and saturation level of all study sequences (**Figure 7A**, **Supplementary Figure S2**). Only 63 ASVs were shared between CON and GE diet groups while the latter generated 548 additional unique ASVs compared to 218 in CON (**Figure 7B**). Consistent with ASVs, species diversity (observed ASVs and Chao index) and Shannon and Simpson diversities reflecting richness and evenness of top abundant taxa were significantly higher in the GE group, compared to CON (**Figure 7C**). Distinct bacterial communities in terms of bacterial presence–absence and relative abundance were observed between CON and GE diet groups in the beta ordination PCoA plot. Significant *R* and *p*-values in PERMANOVA indicate the influence of dietary GE in the gut bacterial diversity in terms of shifting in relative abundance of top abundant taxa and augmenting rare bacterial communities compared to the CON group (**Figures 7D, E**). The Bray–Curtis distance showed more balance variation in abundance for bacterial communities in the GE diet compared to CON feed (**Figure 7F**).

### 3.6 Gut microbial composition

**Figure 8:**
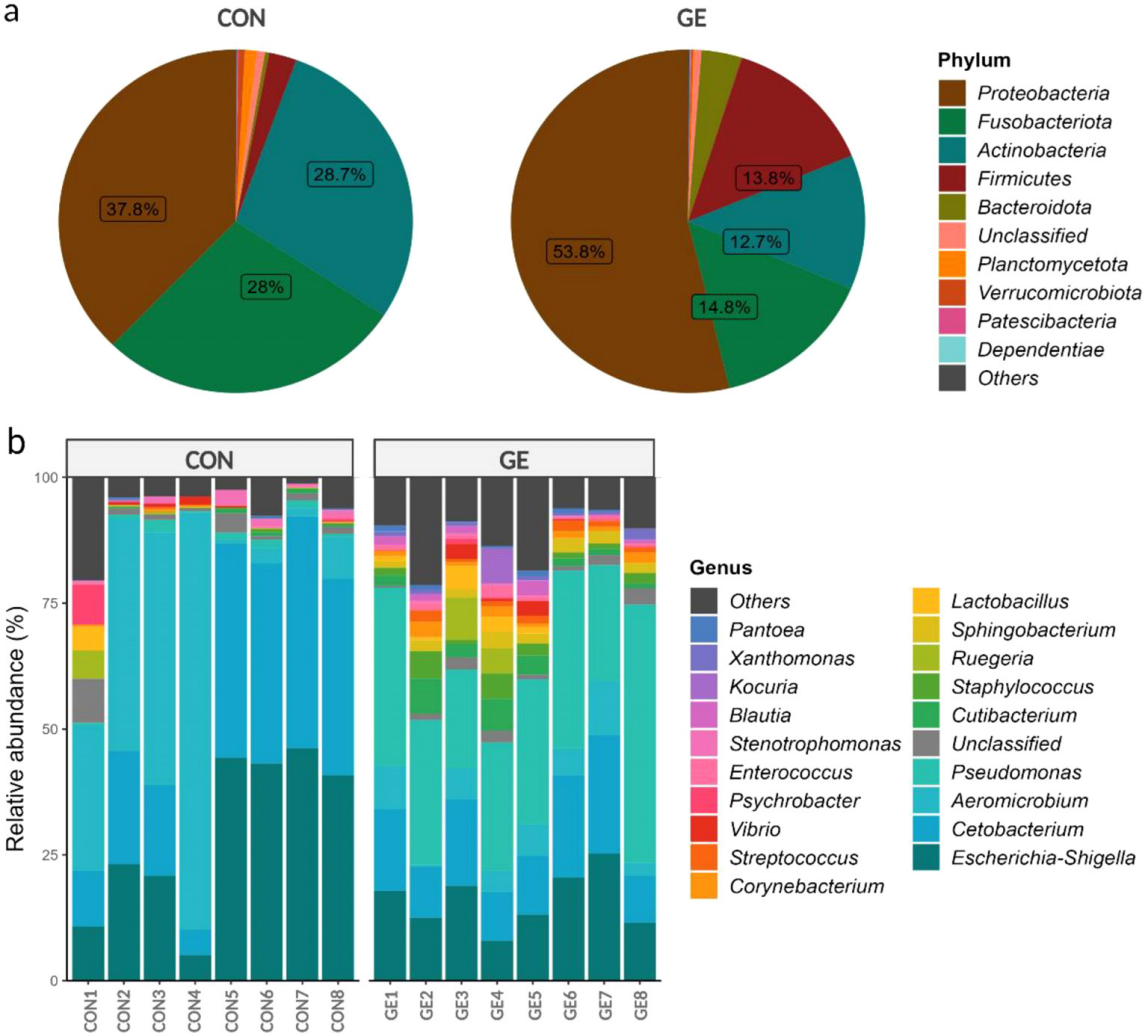
Relative abundance (≥1%) of bacteria in the hind gut of Nile tilapia. **(A)** Pie chart representing gut bacteria at the phylum level. **(B)** Bar plot representing gut bacteria at the genus level. GE, *Gracilaria* extract; CON, control.

**Figure 9:**
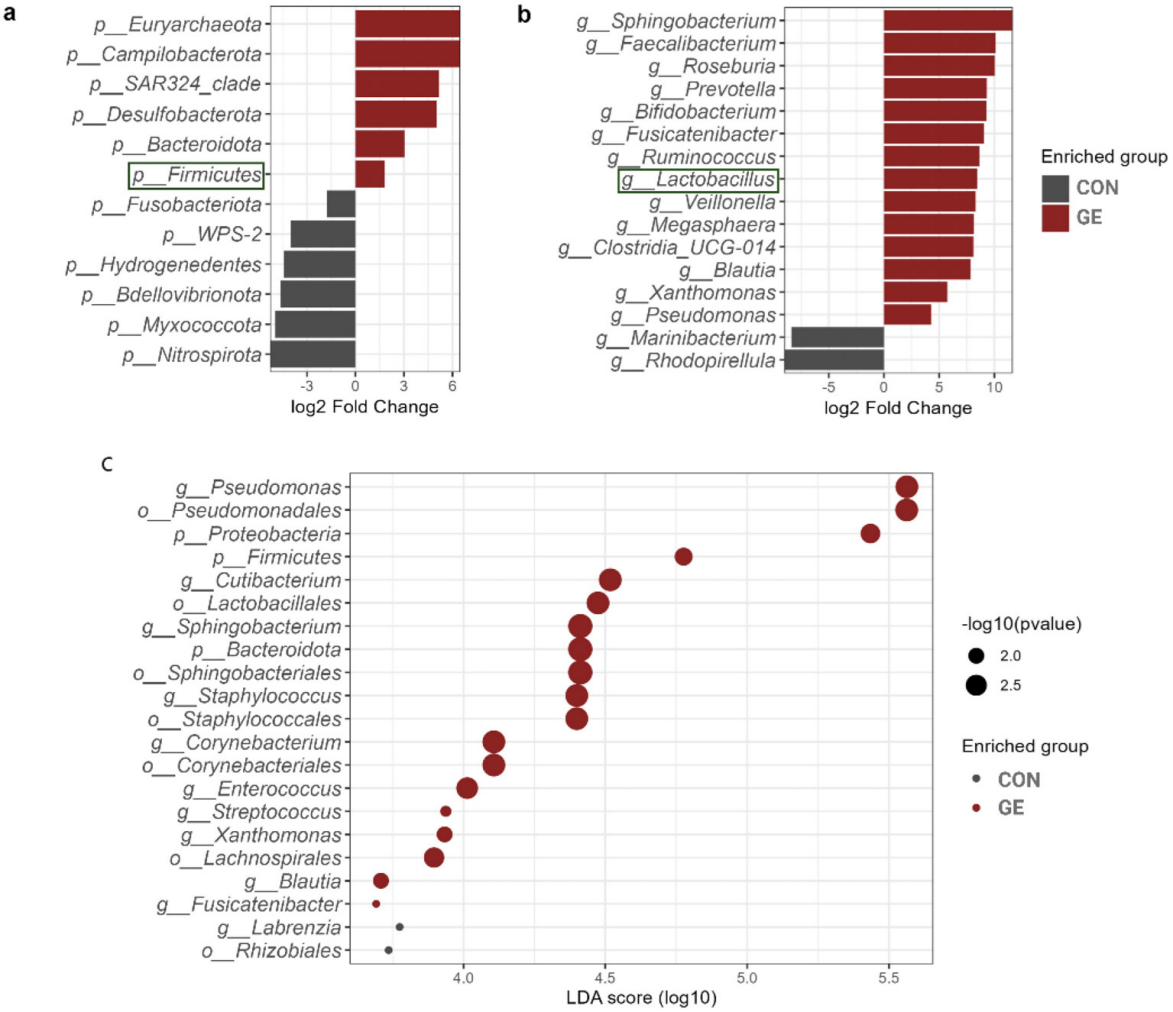
Differential abundant taxa in the hind gut of Nile tilapia. **(A)** Comparison of different phyla in the two dietary groups of GE and control. **(B)** Comparison of different genera in the two dietary groups of GE and control. The health beneficial genus is marked with green color. **(C)** Enriched phyla, order, and genera with log10 *p*-value. The *X*-axis indicates the LDA score with dots representing the significance level of *p*-values. Bigger dots indicate more significant *p*-value. GE, *Gracilaria* extract; CON, control.

**Figure 10:**
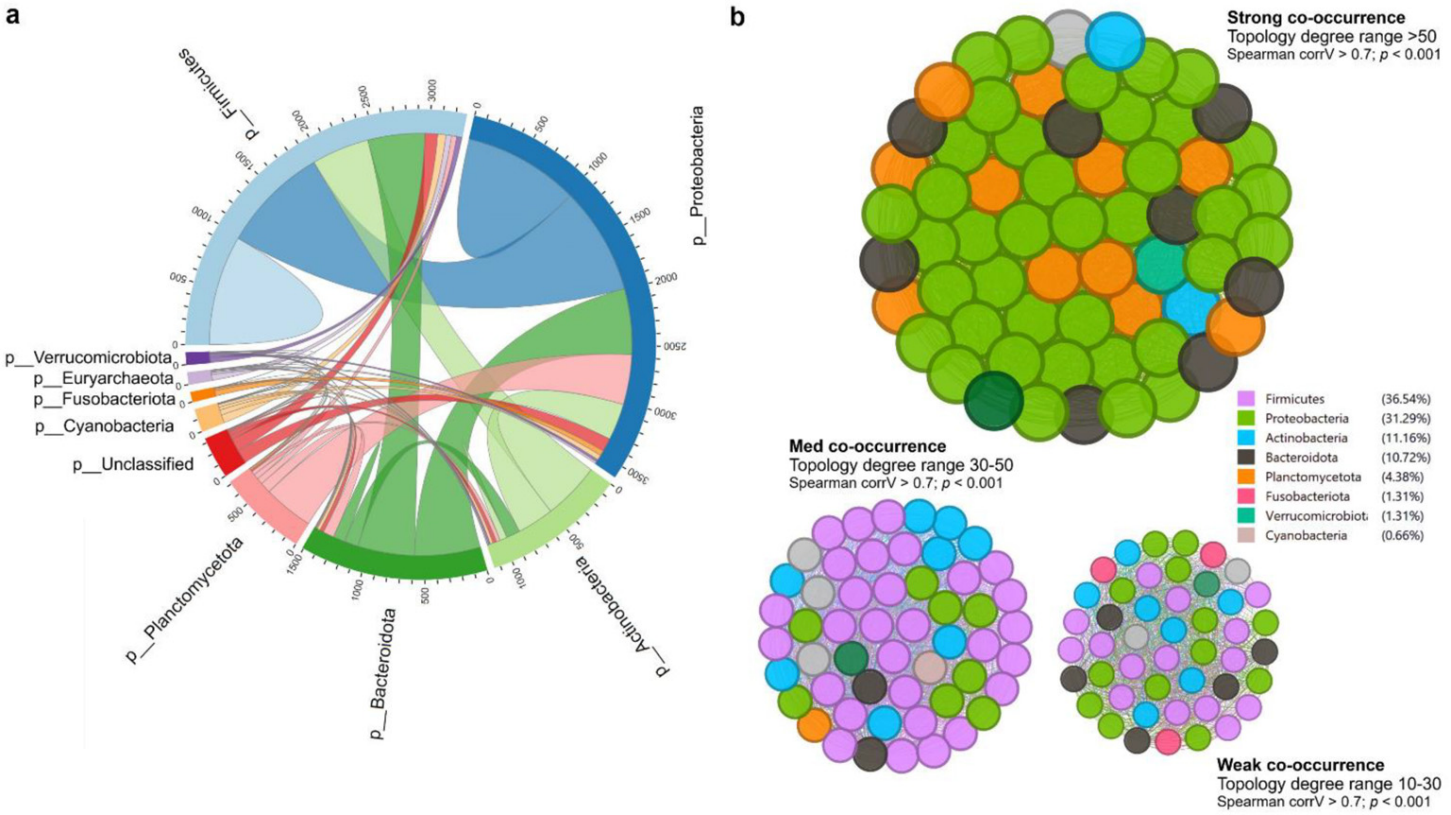
Microbial interaction and co-occurrence network. **(A)** Cladogram representing the correlation of amplicon sequence variants (ASVs) between two diets in terms of interactions. The number in the outer circular part indicates the average ASV counts per sample. **(B)** Co-occurrence network of bacterial community regarding the degree of interactions. The network was segregated based on topology wherein the size of the nodes represents the degree of interaction (10%–100%). The color code on the right side indicates the phyla and contributions to the interaction.

At the phylum level, Proteobacteria represented approximately half of the classified reads (45%) in both groups —37.8% in CON and 53.8% in GE, while Fusobacteriota (28%) and Actinobacteria (28.7%) composed 56.7% of reads in the CON diet. Firmicutes (13.8%) had a higher abundance than Fusobacteria (14.8%) and Actinobacteria (12.7%) in the fish gut-fed GE diet. Firmicutes abundance was only 2.6% in the CON diet, indicating the influential impact of GE diet on this phylum. Alongside Firmicutes and Bacteroidota, abundance also increased from <0.5% in CON to 3.8% in the GE diet (**Figure 8A**). At the genus level, *Aeromicrobium*, *Escherichia-Shigella*, and *Cetobacterium* comprised 84.6% of the total reads in fish fed the CON diet while *Pseudomonas* (32.3%) was the most abundant bacterial group in fish fed the GE diet, followed by *Escherichia-Shigella* (14.5%), *Cetobacterium* (13.3%), *Aeromicrobium* (4.9%), *Cutibacterium* (3.6%), and *Sphingobacterium* (2.2%) (**Figure 8B**). Dietary GE provision significantly promoted the abundance of Bacteroidota, Campilobactorota, Desulfobacterota, and Firmicutes at the phylum level compared to Nitrospirota in the CON diet (**Figure 9A**). At the order and genus level, *Sphingobacterium*, *Prevotella*, *Lactobacilliles*, *Lactobacillus*, *Roseburia*, *Bifidobacterium*, and *Ruminococcus* showed significantly higher abundance in the GE diet compared to *Rhodopirellula* and *Rhizobiales* in CON (**Figure 9B**, **Supplementary Figure S3**). The GE diet also increased abundance for some opportunistic pathogens including *Streptococcus*, *Staphylococcus*, and *Corynebacterium* (**Figure 9C**). A shift in bacterial abundance in the gut also modulates metabolic pathways in the dietary GE group, specifically amino acid biosynthesis and metabolism compared to glucose–sucrose metabolism in the CON group (**Supplementary Figure S4**). The microbial interaction based on ASV correlation showed the dominance of Proteobacteria and Firmicutes in the network. Some of the Proteobacteria and Firmicutes communities were found to be self-interactive (**Figure 10A**). Despite their high abundance, Actinobacteria and Fusobacteria were less interactive. Microbial co-occurrence network analysis revealed that Firmicutes led interactions in the community, despite the Proteobacteria-rich environment and their dominance in strong interactions. Firmicutes were associated with over 70% of medium and 50% of weak interactions, mostly with Bacteroidetes and Actinobacteria. Proteobacteria led the strong interactions and showed no involvement with Firmicutes (**Figure 10B**).

## 4 Discussion

Extreme temperature events caused by climate change have substantial impacts on aquatic ecosystems and can affect the aquaculture industry in various ways. These include changes in water temperature, reduced dissolved oxygen levels, and facilitating the spread of pathogens and parasites in farming environments (2). These challenges lead to increased stress and disease susceptibility and reduced growth rates in fish. Sustainable and responsible aquaculture practices, coupled with a broader commitment to mitigating temperature-induced stress, are essential for ensuring the resilience of fish farming in the face of global warming. Nutritional modulation and enrichment of aquafeeds can play a crucial role in mitigating the negative effects of acute temperature stress on aquaculture systems. Nowadays, the application of seaweeds and seaweed -based functional metabolites in aquafeeds has greatly attracted worldwide attention, demonstrating positive effects on stress reduction and improved disease resistance and overall health (14, 41). The findings of the present study demonstrate the potential of using GE in aquafeeds to counteract stress and improve growth performance while rearing at high temperature via the modulation of antioxidant status and gut microbiota composition in Nile tilapia.

Fish fed the GE-supplemented diet in the present study significantly improved the growth performance and feed utilization. These findings align with previous studies that have demonstrated the positive effects of incorporating seaweeds such as *Gracilaria persica* into fish diets, benefitting the growth performance of Atlantic salmon (42) and Persian sturgeon, *Acipenser persicus* (43), respectively. The improved growth realized in the present study may be due to the availability of nutrients (polyunsaturated fatty acids, favorable amino acids, minerals, and vitamins) and bioactive metabolites (phenolic, flavonoid, and β-carotene) in the seaweed extract that stimulated the digestion and absorption by fish (44, 45). Furthermore, the growth increment shown in this study may also be attributed to the presence of large amounts of polysaccharides and oligosaccharides present in seaweeds that act as prebiotics, thus positively increasing the digestion and assimilation of nutrients by enhanced activity of the beneficial bacteria (46). Moreover, the growth of fish has a close connection with the development of muscle that is attributed to its hyperplastic and hypertrophic fiber production (47, 48). Quantitative observations of muscle fiber morphology have been considered to be essential to evaluate muscle growth in nutritional research. Production of new muscle (hyperplasia) and the increment of existing muscle (hypertrophy) have been greatly stimulated by dietary composition (47). The increment of size and consequent decrease in the number of muscle fibers of Nile tilapia fed the GE diet in this study revealed the positive impacts of dietary GE, assisting in growth enhancement. In the current study, the FCR of Nile tilapia significantly improved due to the dietary supplementation of GE. This result is consistent with the study on Indian major carp, *Labeo rohita*, fed fucoidan-rich *Sargassum wightii* extract (49). The improved FCR could be attributed to the enhanced feed palatability and feed intake, which may have resulted from the activity of phenolic and flavonoid bioactive compounds in seaweed (50). Moreover, these biologically active compounds stimulate the secretion of important digestive enzymes such as amylase, lipase, and protease that significantly improve digestion and improve feed utilization (51).

The liver is an important organ that regulates the metabolism of essential nutrients and controls energy storage and utilization to maintain homeostasis (52). Aquafeeds high in fat and carbohydrates can lead to an increase in liver fat as well as larger adipocyte size (hypertrophy), causing the accumulation of body fat, while optimum feeding and proper nutrition can reduce adipocyte size (atrophy) as excess fat is utilized for energy (53). Therefore, understanding liver health and adipocyte tissue and their regulation is essential in studying dietary nutrition and related metabolic issues in fish. The significant reduction in liver fat coupled with the reduction of adipocyte size in fish fed the GE diet could be related to the reduction of AST and ALT enzyme activity levels compared to the control diet. This reduction in liver fat and adipocyte size in GE-fed fish may also be related to the abundance of intestinal microbiota, which may influence fat absorption by producing metabolites such as short-chain fatty acids and secondary bile acids, as well as pro-inflammatory bacterially derived factors such as lipopolysaccharides (54, 55). However, more research is needed to gain a comprehensive understanding of how GE affects liver lipid accumulation and adipose tissue quantity and size in fish.

Lysozyme activity is a vital component of fish innate immune system to defend against infections (56). The current study revealed higher lysozyme activity in fish fed GE, suggesting a boosting immunity possibly triggered by active compounds including polyphenols and polysaccharides present in *Gracilaria* sp. (57). Moreover, this finding can be attributed to the seaweed’s role in activating the immune response and scavenging excessive reactive oxygen metabolites (ROS). In accordance with the current study, the administration of *Gracilaria* sp. extract increased lysozyme activity in European seabass (58). Fish under stressful conditions often experience oxidative stress, which is caused by an imbalance between the production and elimination of ROS within cells (59). The disruption of scavenging capacity results in lipid peroxidation, ROS buildup, and rupturing the lipid membrane of body cells (60). The DNA of immune cells is likewise harmed by this interruption. In this instance, the body secretes SOD, CAT, and GPx, which are enzymatic and non-enzymatic antioxidative responses that reduce ROS and increase antioxidative capacity (61). However, failing to do so leads to elevated lipid peroxidation and the generation of MDA (62). The current study revealed that dietary GE-based diet significantly reduced the MDA level in fish confirming the stress-inhibitory role of dietary GE. These results align with other studies that have found dietary supplementation of *Sargassum horneri*, and a mixture of seaweed (i.e., *Ulva lactuca*, *Jania rubens*, and *Pterocladia capillacea*) extract induced significantly lower MDA levels in black sea bream, *Acanthopagrus schlegelii*, and striped catfish, *Pangasianodon hypophthalmus* (63, 64). The ameliorative capacity of the seaweed extract was associated with the high content of bioactive substances such as flavonoids and phenolic substances and could act as natural antioxidants and play an important role to neutralize free radicals and improve stress resilience (65, 66). In line with the present results, we also observed an increased upregulation of tight junction proteins and antioxidant gene in the hind gut of GE-fed Nile tilapia. These data suggested that GE supplementation protects gut permeability and translocation of pathogens in fish while improving nrf2 pathway and strengthening antioxidant defense. Likewise, in the present study, *hsp70* protein expression was downregulated in the GE group compared to the CON group, indicating stress-mediating effects of GE in fish under adverse conditions.

RBCs play a crucial role in oxygen transport throughout the body, and an increase in their count can lead to higher levels of haemoglobin (67). This allows fish to efficiently transport oxygen from the gills to various tissues and organs. As a result, fish are likely to cope better with stressors, such as changes in water temperature, pollution, or other environmental challenges. In the present study, a significant increase in RBC levels was found in fish fed a GE diet. Similar findings have been observed in striped catfish, where a mixture of seaweeds (*U. lactuca*, *J. rubens*, and *P. capillacea*) increased the RBC count (63). Another study showed that a *Sargassum angustifolium* algae extract-supplemented diet enhanced the number of RBCs in rainbow trout (68). These results suggest that supplementing the diet with seaweed may enhance erythropoietin production and erythrocytic stability, which may increase the RBC level in fish (69). Furthermore, fish fed the GE diet in the present study demonstrated a significant decrease in RBC cellular and nuclear abnormalities, indicating the beneficial effects of seaweed in mitigating stress during extreme warm exposure. Warm stress generally results in transmembrane alterations and metabolic inhibition that trigger the DNA-damaging mitochondrial caspase-3, which leads to erythrocytic cellular and nuclear anomalies (70–73). Furthermore, elevated water temperatures can also damage DNA by causing the release of DNase enzymes from lysosomes and thermally inactivating the enzymes that repair DNA, which can change the shape of cells (74). Significantly higher RBC cellular and nuclear abnormalities observed in fish fed the control diet could be due to the excess lipid peroxidation that considerably increased the permeability and decreased the symmetry of the erythrocyte cell membrane, which is also responsible for higher abnormalities in erythrocytes (75). Nevertheless, the higher RBC abnormalities with the control diet are justified by the much higher overexpression of *hsp70* identified in these fish when compared to GE-fed fish.

The present study found that Nile tilapia fed a diet supplemented with GE exhibited a healthier gut, as indicated by the higher diversity and abundance of bacterial populations and enrichment of phylum Firmicutes, family *Lactobacillales*, and genus *Lactobacillus*. These bacteria are mostly considered beneficial for fish and other aquatic animals (76). A study by Cui et al. (77) has shown that supplementing the diet with fucoidan, a polysaccharide extracted from brown-seaweed *Undaria pinnatifida*, enhanced the activity of digestive enzymes and regulated the microbial populations and increased beneficial bacteria in fish. The increased abundance of *Lactobacillus* is of considerable interest since an enrichment in these bacteria is associated with improved health (76). The increased bacterial diversity and higher number of *Lactobacillus* in Nile tilapia fed GE may be due to the presence of bioactive compounds (e.g., short-chain peptides, polyphenols, and polysaccharides) in seaweed, which might have induced the colonization of health-promoting bacteria. Polysaccharides in seaweed are a preferential substrate for lactic acid bacteria (78), as demonstrated in the gut of zebrafish, *Danio rerio* (79), and barramundi, *Lates calcarifer* (80). Additionally, *Lactobacillus* has been linked to various protective mechanisms in the host against pathogens, such as producing bacteriocin to remove pathogens from the gut epithelium (81). In the present study, feeding fish with seaweed extract decreased the abundance of opportunistic pathogen *Escherichia-Shigella* (*p* < 0.05, LDA < 2.0) in Nile tilapia gut. This result is supported by *Sargassum dentifolium* extract that reduced the abundance of *Escherichia coli* in the gut of king prawn, *Litopenaeus vannamei* (82). Studies indicate that increasing environmental temperatures have adverse effects on fish, leading to reduced digestive efficiency, lower gut bacterial abundance, and higher pathogenic bacteria with negative implications for the host’s health and physiology (83, 84). Despite the augmentation of Firmicutes and *Lactobacillus*, GE also influenced the colonization of some opportunistic clinical pathogens including *Streptococcus*, *Staphylococcus*, and *Corynebacterium* that needs further investigation. Nevertheless, the higher diversity of bacteria in the GE-fed group compared to the control group indicates the effects of seaweed even at high temperatures. Future research should focus on understanding the impact of temperature variations on the fish microbiome and the consequences of microbial changes on fish performance.

## 5 Conclusion

**Figure 11:**
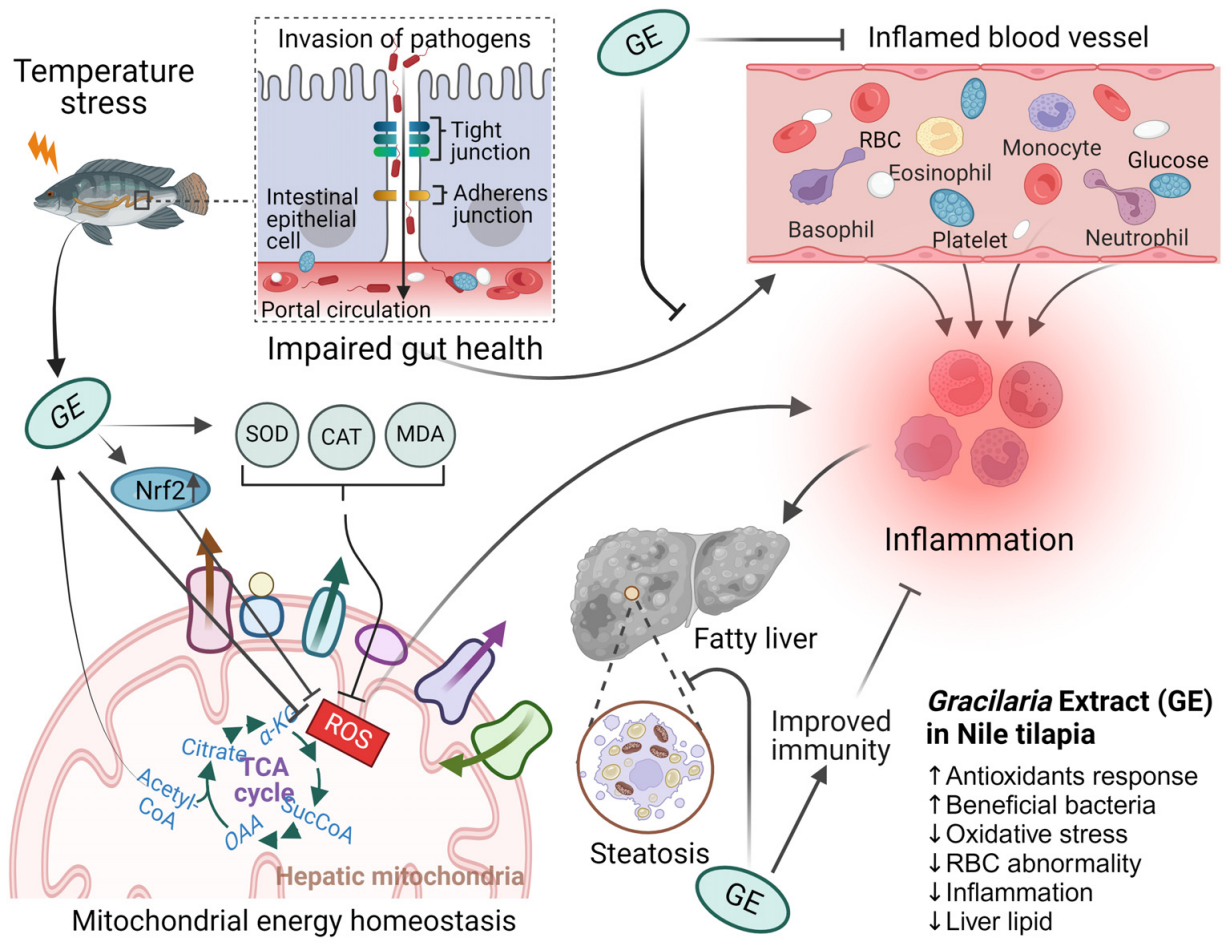
The possible roles of *Gracilaria* extract (GE) on the gut–liver axis and its underlying mechanisms in Nile tilapia. Nile tilapia under acute temperature stress is susceptible to pathogen invasion through a leaky gut caused inflammation that compromises immune function of fish. Dietary supplementation with GE increases the expression of antioxidant gene (*nrf2*) and reduces MDA level, which leads to improved mitochondrial function and energy production to help prevent inflammation and subsequent oxidative stress in fish. The GE diet has also been found to reduce liver lipid content, an indication of improved health of fish. The figure includes up and down arrows, denoting increased and decreased cellular functions and systems, respectively.

This study demonstrates that dietary *Gracilaria* extract (1% of feed) supplementation could counteract the adverse effects of temperature-induced oxidative stress in fish through (1) enhancing the antioxidant capacity indicated by the upregulation of *nrf2* expression and reduction of MDA production; (2) protecting gut permeability by improving the expression of tight junction proteins (*occludin* and *claudin1*); (3) increasing the RBC count, which could indicate a higher blood flow in the circulatory system; (4) reduction of stress levels indicated by the reduction of *hsp70* mRNA expression; and (5) increasing the number of potentially health-promoting bacteria in fish gut. A summary of the beneficial role of seaweed extract in the gut–liver axis and its underlying mechanisms is depicted in **Figure 11**. The present findings reinforce the prospect of seaweed extracts as an effective supplement in aquafeeds to attenuate temperature-induced stress in aquaculture production during summer in the face of global warming.

## Data Availability

The datasets presented in this study can be found in online repositories. The names of the repository/repositories and accession number(s) can be found in the article/**Supplementary Material**.
