# Supplementary material for: Dietary seaweed extract mitigates oxidative stress in Nile tilapia by modulating inflammatory response and gut microbiota
Source: Front Immunol. 2024 Nov 21;15:1471261. doi: 10.3389/fimmu.2024.1471261 (PMC11617724; doi:10.3389/fimmu.2024.1471261)
Supplement: Supplementary file 1 [file DataSheet1.docx]

**Supplementary Files**

**Figure S1**


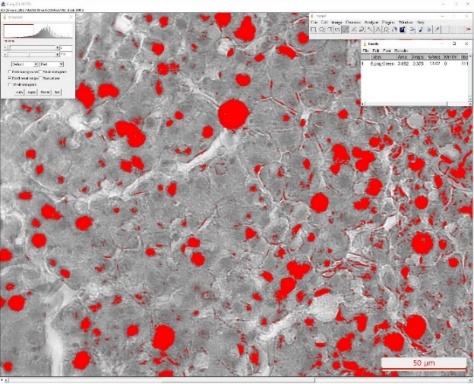

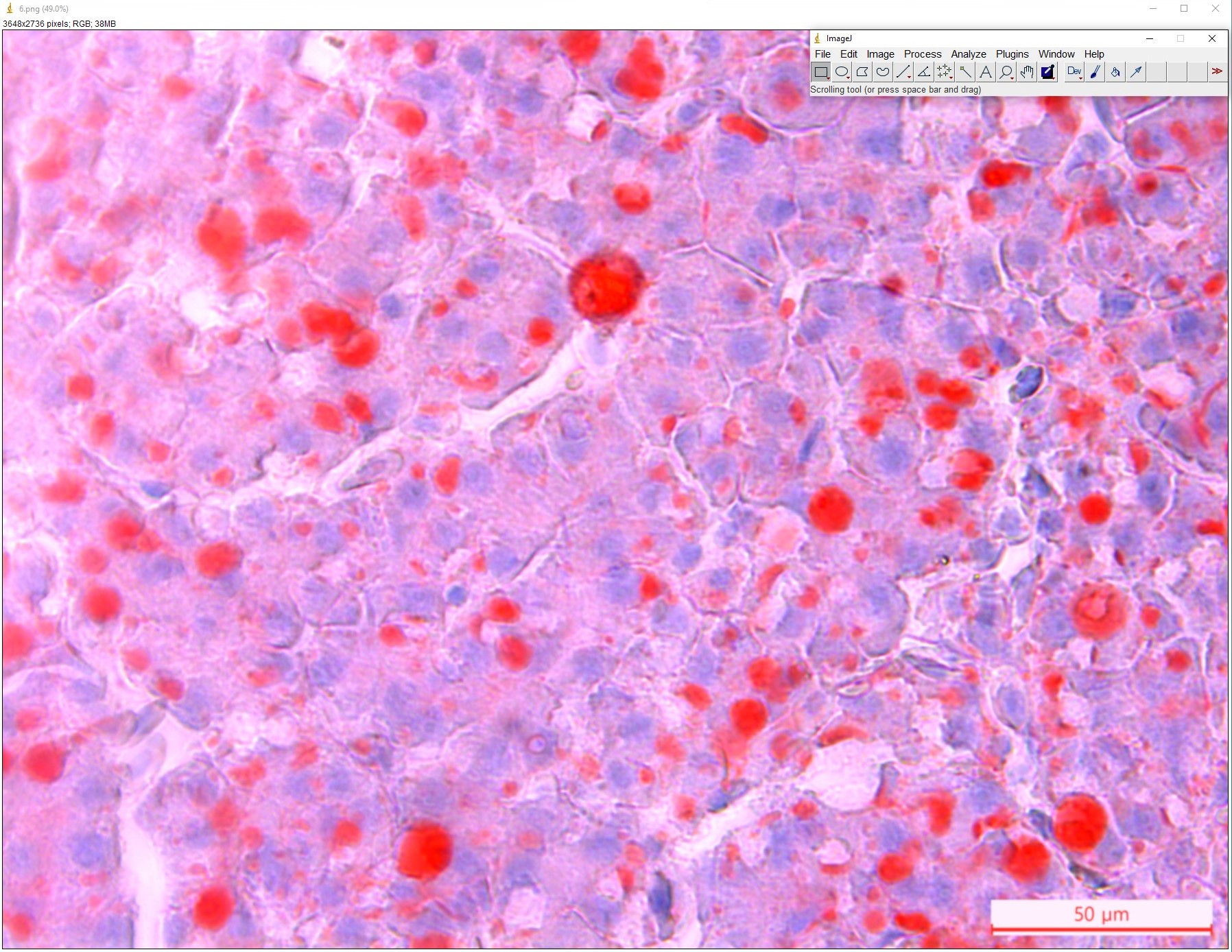

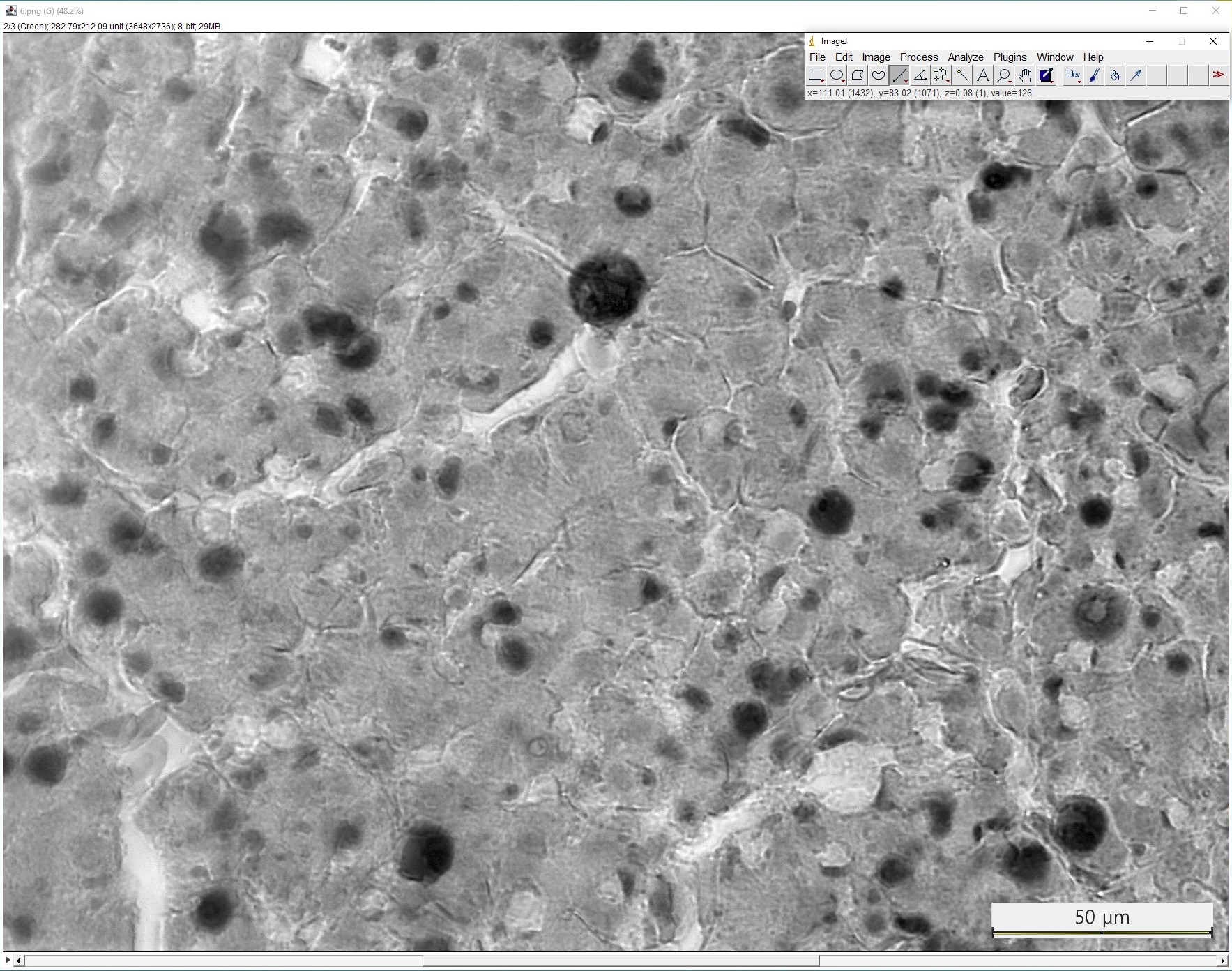

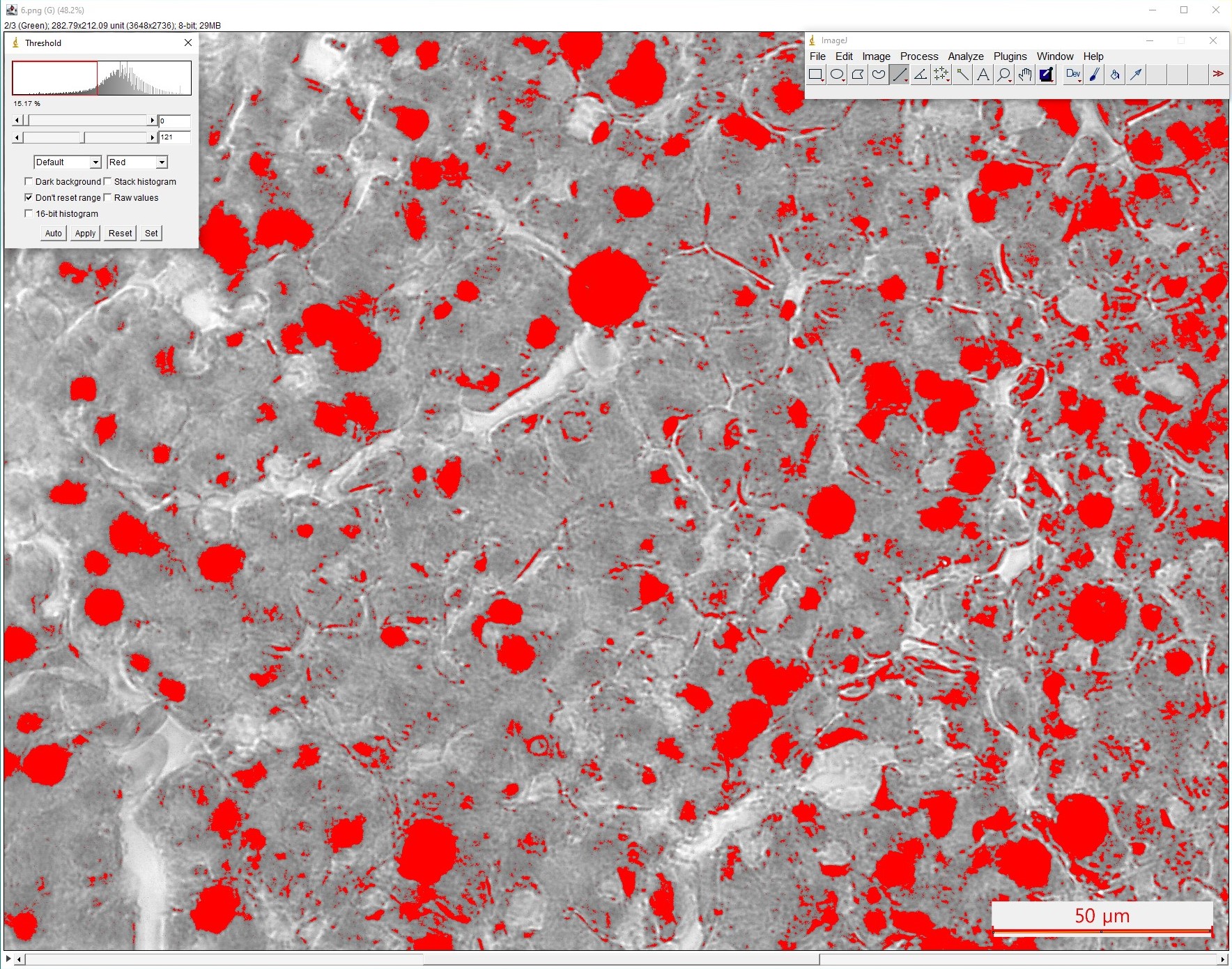


**a**

**b**

**c**

**d**

**Figure S1**. Estimation of lipid droplet accumulation in the liver. The neutral lipid droplets in the liver tissue of Nile tilapia were assessed using Oil Red O (ORO) and H&E staining. Random images were captured using a 20X microscopic lens. The captured images were then adjusted using ImageJ software (version 1.53). To adjust the images, went to the menu bar and selected 'Image' and 'Type', followed by 'RGB Stack' in the toolbox. Next, selected 'Image' in the menu bar and chose 'Stacks', then 'Make Montage' in the toolbox. In the 'Make Montage' toolbox, needed to make sure that 'Columns' is set to 3 in order to create the Montage. Then, went back to the menu bar, select 'Image', followed by 'Adjust', and finally 'Threshold'. Adjusted the color according to the visibility of the neutral lipid droplets and click 'set' to establish the 'Lower and Upper Threshold levels'. Once the color threshold of the image has been adjusted, selected 'Analyze' and 'Set Measurements' in the toolbox. In the 'Set Measurements' toolbox, chose 'Area', 'Area fraction', 'Limit to threshold', and 'Display label' respectively. Lastly, selected 'Analyze' in the menu bar and click on 'Measure' in the toolbox to obtain the (%) results of neutral lipid droplets in the liver tissue. [**a-b**, representative H&E-stained liver histology image; **b-c**, Oil Red O-stained liver histology image].

**Figure S2**


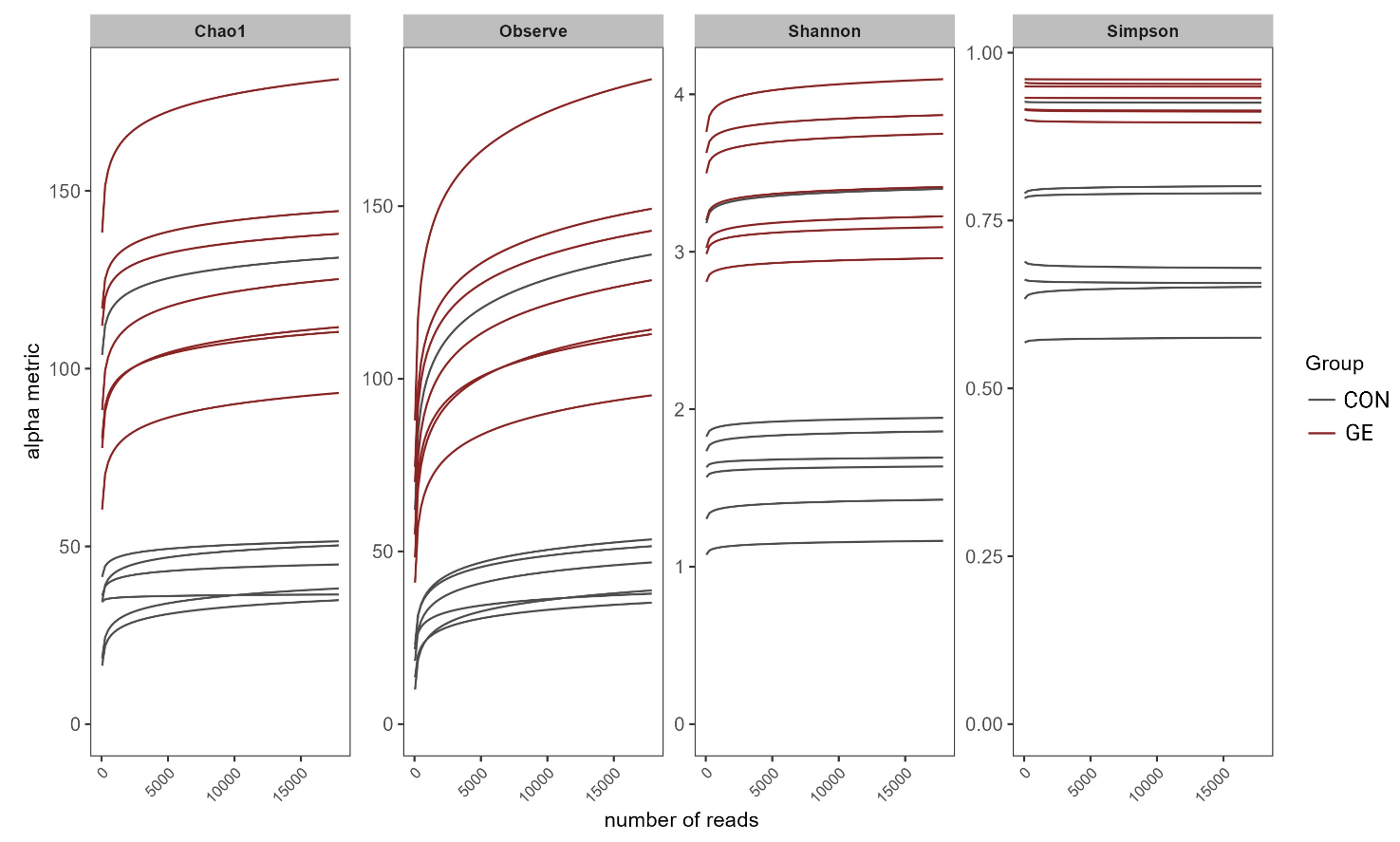


**Figure S2**. Details of rarefaction curve in terms of alpha-diversity – Chao1, Observed, Shannon, and Simpson indices. X-axis indicates number of reads and Y-axis represents alpha metric values. CON, control; GE, *Gracilaria* extract.

**Figure S3**


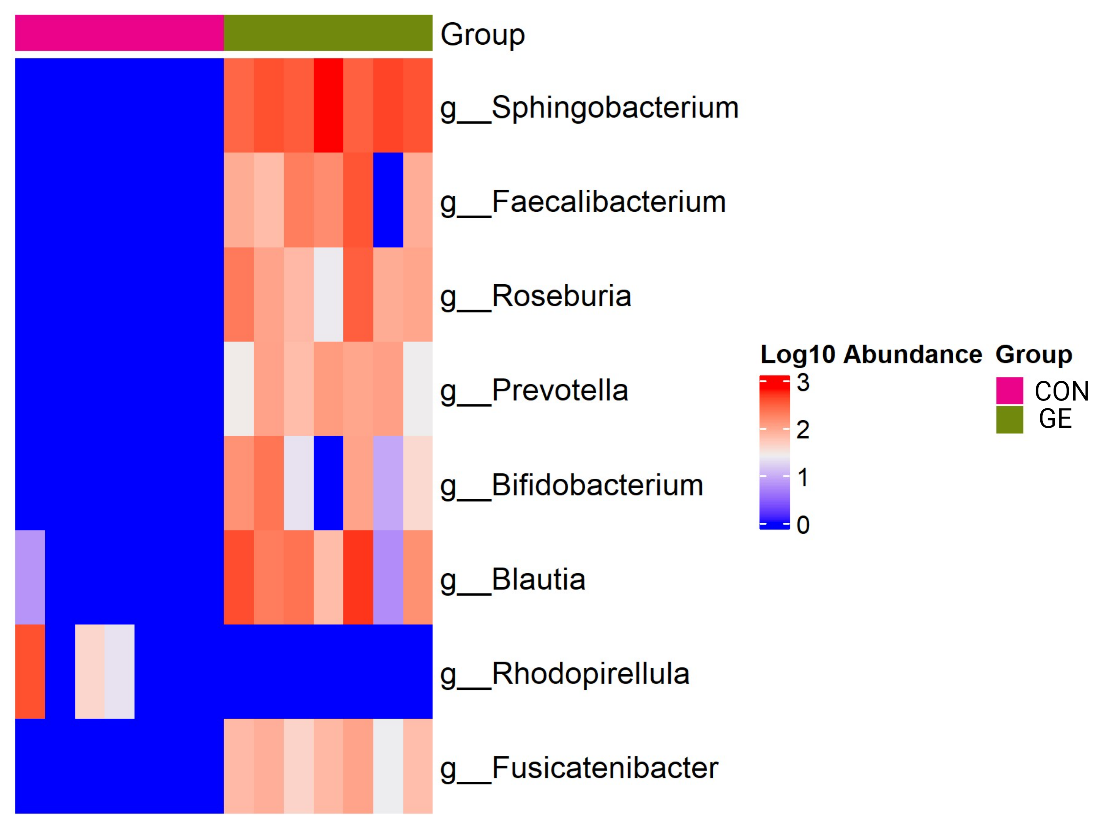


**Figure S3**. Heatmap showing replicate-specific differential abundance between CON and GE groups. CON, control; GE, *Gracilaria* extract.

**Figure S4**


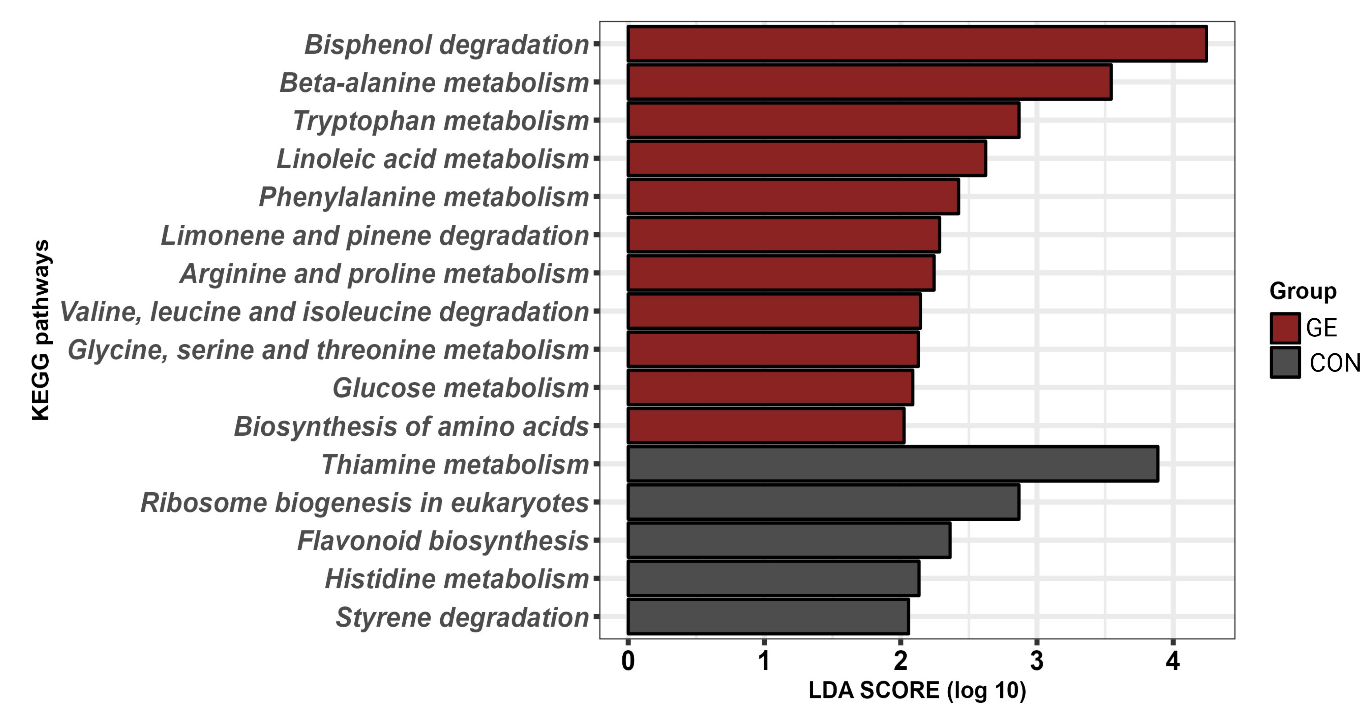


**Figure S4**. Metagenome prediction of functional pathways enriched after 70-day feeding trial. CON – control; GE, *Gracilaria* extract.
